# Nurse-led hospital violence intervention programme versus standard care in Wales: a cost-effectiveness analysis

**DOI:** 10.1016/j.eclinm.2026.104108

**Published:** 2026-07-24

**Authors:** Shainur Premji, Simon C. Moore, Daniel Tod, Megan Hamilton, Amrita Bandyopadhyay, Jonathan Shepherd, Henry Yeomans, Adele Battaglia, David O'Reilly, Simon M. Walker

**Affiliations:** a Centre for Health Economics, University of York, York, YO10 5DD, United Kingdom; b Violence Research Group, School of Dentistry, Cardiff University, Heath Park, Cardiff, CF14 4XY, United Kingdom; c Security Crime & Intelligence Institute, Cardiff University, PO Box 1182, Cardiff, CF11 1AZ, United Kingdom; d York Trials Unit, University of York, York, YO10 5DD, United Kingdom; e Centre for the Improvement of Population Health Through e-Records Research (CIPHER), Swansea University, Swansea, SA2 8PP, United Kingdom; f Public, Patient Involvement Lay Member, United Kingdom; g Cardiff Liver Unit, Cardiff and Vale NHS Trust, Cardiff, CF14 4HH, United Kingdom; h Academic Department of Military Surgery and Trauma, Defence Medical Services, Birmingham, B15 2WB, United Kingdom

**Keywords:** Violence, Emergency department, Prevention, Public health, Cost-effectiveness

## Abstract

**Background:**

Violence is a major public health issue. In the United Kingdom (UK), injuries resulting from interpersonal violence were estimated to cost the National Health Service (NHS) £2.9 billion annually. Hospital Violence Intervention Programmes (HVIPs), based in Emergency Departments (EDs), identify individuals (both perpetrators and victims) who may benefit from support for modifiable risk factors associated with exposure to violence. In this study we evaluate the cost-effectiveness of implementing an HVIP called the Violence Prevention Team (VPT) relative to standard care in Wales.

**Methods:**

We conducted a cost-effectiveness analysis of VPTs in ED relative to standard care. We included patients aged 11 years or older who attended ED with an assault-related injury between November 2019 and February 2024 in Wales. Participants exposed to the VPT or standard care were matched with a minimum 1:1 ratio. A hybrid decision tree-Markov model was created to follow patients attending ED for an initial assault-related attendance and to track any subsequent unplanned ED visits thereafter. Health outcomes were measured in quality-adjusted life years (QALYs), and costs (in 2023/2024 British pounds sterling) were estimated using a (a) health perspective and (b) societal perspective (including additional costs across the third sector) over a time horizon of 10 years. Routine data from the Secure Anonymised Information Linkage (SAIL) databank were used to inform model probability and healthcare cost estimates. QALYs were estimated using the literature. We estimated the incremental cost-effectiveness ratio (ICER), net monetary and net health benefits, and probability of being cost-decreasing and QALY-increasing. ICERs were interpreted against the UK Department for Health's cost effectiveness threshold of £15,000 per QALY and the National Institute for Health and Care Excellence (NICE) cost-effectiveness threshold of £20,000–£30,000 per QALY. This study is registered with ISRCTN (68945844).

**Findings:**

Our base case analysis suggested that implementing VPTs in ED was cost-saving and more effective relative to standard care, from both a health and societal perspective. At a NICE cost-effectiveness threshold of £20,000–£30,000 per QALY, there was 83% probability that VPTs in ED are considered cost-effective relative to standard care. At a threshold of £15,000 per QALY, the probability that VPTs in ED are considered cost-effective relative to standard care was 84%. When only including ED and associated inpatient admission costs and excluding primary care visit costs, VPTs remained cost-effective from a health perspective (£12,950 per QALY gained).

**Interpretation:**

VPTs in ED are strategically placed to intervene following injuries. They have the potential to reduce costs and improve health outcomes, and their implementation should be prioritised by the NHS.

**Funding:**

This study was funded by the 10.13039/501100000272National Institute for Health and Care Research, Public Health Research Board (NIHR134055).

**Research in context**

**Evidence before this study**

Approximately 84% of injury-specific short-term health-related costs in Wales are associated with interpersonal violence. Despite an overall reduction in the levels of violence in England and Wales, gun and knife-related assaults and homicides have increased.

We searched PubMed, MEDLINE, and Google Scholar from database inception to March 2024, for papers published in English, using search terms that primarily included “violence”, “injury”, “effectiveness”, “cost”, “cost effectiveness”, “health-related quality of life” and variations thereof, which resulted in 25 papers being retained for full text review. Existing evidence for hospital-based violence intervention programmes (HVIPs) suggests they may provide a cost-effective solution. However, variations in operationalisation and the broader health and public sector environments within which they operate make it challenging to generalise to the United Kingdom (UK) context.

To our knowledge, this is the first study to evaluate the cost-effectiveness of an HVIP called the Violence Prevention Team (VPT) relative to standard care in the UK. Previous research on this programme has demonstrated the VPT is more effective at reducing subsequent unplanned emergency department (ED) attendances relative to standard care.

**Added value of this study**

VPTs are based in EDs and VPT nurses and advocates work with clinical staff to identify whether patients attending ED have been exposed to interpersonal violence. Once identified, risk- and needs-based assessments determine whether patients would benefit from additional support targeting any underlying vulnerabilities. Our findings demonstrate that implementing VPTs in ED have the potential to save health system costs and improve health outcomes for patients affected by violence relative to standard care. Cost savings were primarily driven by the effectiveness of the VPTs in reducing subsequent unplanned ED attendances and related hospitalisations. Our base case findings were consistent when using both a health and a societal perspective.

**Implications of all the available evidence**

HVIPs are strategically placed to intervene following injuries from violence. From a health perspective, the evidence is strong that nurse-led VPTs are cost-saving and improve health outcomes. If the UK were to implement VPTs at scale, this would result in a relative reduction of just over 7500 unplanned ED attendances and a cost savings of £102 million for the National Health Service. Improving the uptake of VPT referrals beyond 35% could further amplify benefits. Accordingly, VPT implementation should be prioritised.

## Introduction

Over the past two decades, overall levels of interpersonal violence have been declining in England and Wales, yet rates of gun violence, knife violence, and homicide have been increasing.^1^ Violence is the intentional threat or use of physical force or power against oneself or others, which frequently results in injury, psychological harm, maldevelopment, death or deprivation.^2^ Violence is a major public health concern and places a heavy health and social burden on individuals over the life course.^2^^,^^3^ Its significant reduction forms part of the United Nations’ 2030 Agenda for Sustainable Development,^4^ and government policies in the United Kingdom (UK) promote whole system multi-agency approaches to interpersonal violence.^1^

In 2008/2009, injuries resulting from violence were estimated to cost the National Health Service (NHS) in England £2.9 billion per year—the equivalent of £4.75 billion today.^5^ Assault-related injuries resulted in over 300,000 annual attendances to the Emergency Department (ED) and over 35,000 hospital admissions.^5^ While these health-related costs were substantial, they represented merely a tenth of the total societal costs,^5^ which additionally included costs related to lost productivity and those incurred by the criminal justice and social welfare systems.^5^^,^^6^

Violence is affected by several risk and protective factors that interact across various ecological levels, from individual choices to family, peer, or local community dynamics, to broader societal and environmental conditions.^6^ These factors have the potential to affect behaviour and increase the likelihood that some individuals will be exposed to interpersonal violence as victims and/or perpetrators.^6^ Similarly, these factors contribute to individual vulnerability to engage in persistent patterns of behaviour, resulting in episodes of recurrent violent injury or repeat victimhood.^7^

Hospital Violence Intervention Programmes (HVIPs), typically situated within Emergency Departments (EDs), have emerged in the UK as a practical way to identify patients attending due to violence and triage them for additional referral and support as required.^8^ In Wales, two intervention Violence Prevention Teams (VPTs) have been introduced, first in Cardiff (est. 2019) and then extended to Swansea (est. 2022).^9^ Full details on these are provided elsewhere.^10^ In brief, the VPT service involves a collaboration between local police services and the NHS, where patients attending ED or referred through other hospital departments or across community healthcare teams (e.g. general practitioners, GPs) are assessed by VPT staff trained to gain an understanding of the circumstances contributing to patients’ exposure to violence. In discharge planning, patients are supported into care pathways (primary, secondary or tertiary care) or third sector organisations, who work with patients to address their modifiable risk factors for violence, with the aim of reducing future recurrent episodes of harm.^8^^,^^9^ Referrals provided by VPTs are additional to existing resources for domestic and sexual violence.^10^ VPT nurses also work within the hospital to train clinical staff on identifying violence-related injury and implementing safeguarding procedures.^10^^,^^11^ Funding to set up these initial VPTs was made available through the UK Home Office, the police-led Violence Prevention Unit in Wales, and the South Wales Police and Crime Commissioner. To date, there has been no rigorous evaluation of this public health approach to violence prevention in the UK, although there is global evidence related to HVIPs, primarily from the United States, that suggests they are beneficial and represent good value for money.^12^^,^^13^

This research forms part of a broader study designed to evaluate the effectiveness and cost-effectiveness of HVIPs in the UK.^10^ Here, we present the cost-effectiveness associated with the implementation of VPTs in ED relative to standard care in Wales. To our knowledge, this is the first cost-effectiveness analysis of HVIPs conducted within a UK context.

## Methods

In this study, we aimed to assess whether the VPT represents value for money, where outcomes were measured in quality-adjusted life years (QALYs) and costs were considered from two perspectives: i) an NHS perspective, and ii) a societal perspective.^14^ Our primary (base case) analysis examined the cost-effectiveness for those who were offered a VPT assessment within an ED setting relative to those who were not (standard care). A *de novo* hybrid decision tree-Markov model was developed to reflect the probabilities, costs, and outcomes associated with subsequent ED attendances and follow-up care. Routine data from the Secure Anonymised Information Linkage (SAIL) databank^15^ were used to inform model probability and healthcare cost estimates, and health-related quality of life (HRQoL) estimates were obtained from the literature. VPT intervention and third sector referral costs (societal perspective only) were also included. Cost-effectiveness results are reported as the incremental cost per QALY, net monetary and net health benefits, and probabilities of being cost-decreasing, QALY-increasing, and cost-effective. A health economic analysis plan was developed for this study.^9^

### Intervention

Eligible patients are either identified by the broader clinical team or by VPT nurses reviewing ED-attending patient records, and their psychosocial vulnerabilities and modifiable risks are ascertained using community and hospital patient records or by the patients themselves. Following identification, VPT nurses work with patients to understand their vulnerabilities and, if suitable, refer them to secondary care (e.g. mental health, drug and alcohol services), third sector (e.g. community, non-profit organisations) or other supports. The aim of the intervention is to target modifiable risk factors for underlying vulnerabilities that are also associated with high intensity use of emergency care in general. Additional details on the intervention and referrals are provided in Supplementary Appendices A1 and A2.

### Standard care

Under standard practice, all clinical staff are expected to have up-to-date safeguarding training and to carry out safeguarding tasks (e.g. refer for additional support). Patients that attend ED with an assault-related injury are treated and encouraged to contact the police or have the ED contact the police on their behalf. If their injury involves weapon use, the ED is obliged to contact the police. ED clinicians can refer patients to special supports if they are suitable (e.g. support for domestic or sexual violence).^9^

### Data linkage and sample

We identified a cohort of eligible individuals using the Welsh Democratic Service, which has complete coverage of all Welsh residents who are registered with a GP. Individuals 11 years of age or older who attended ED with an assault-related attendance during the period November 2019 to February 2024 in Wales were eligible for this study. Those under the age of 11 years were excluded as they are typically supported by paediatric emergency care physicians. Technical details for how the sample was derived is provided in the study protocol^9^ and briefly, below.

**Fig. 1:**
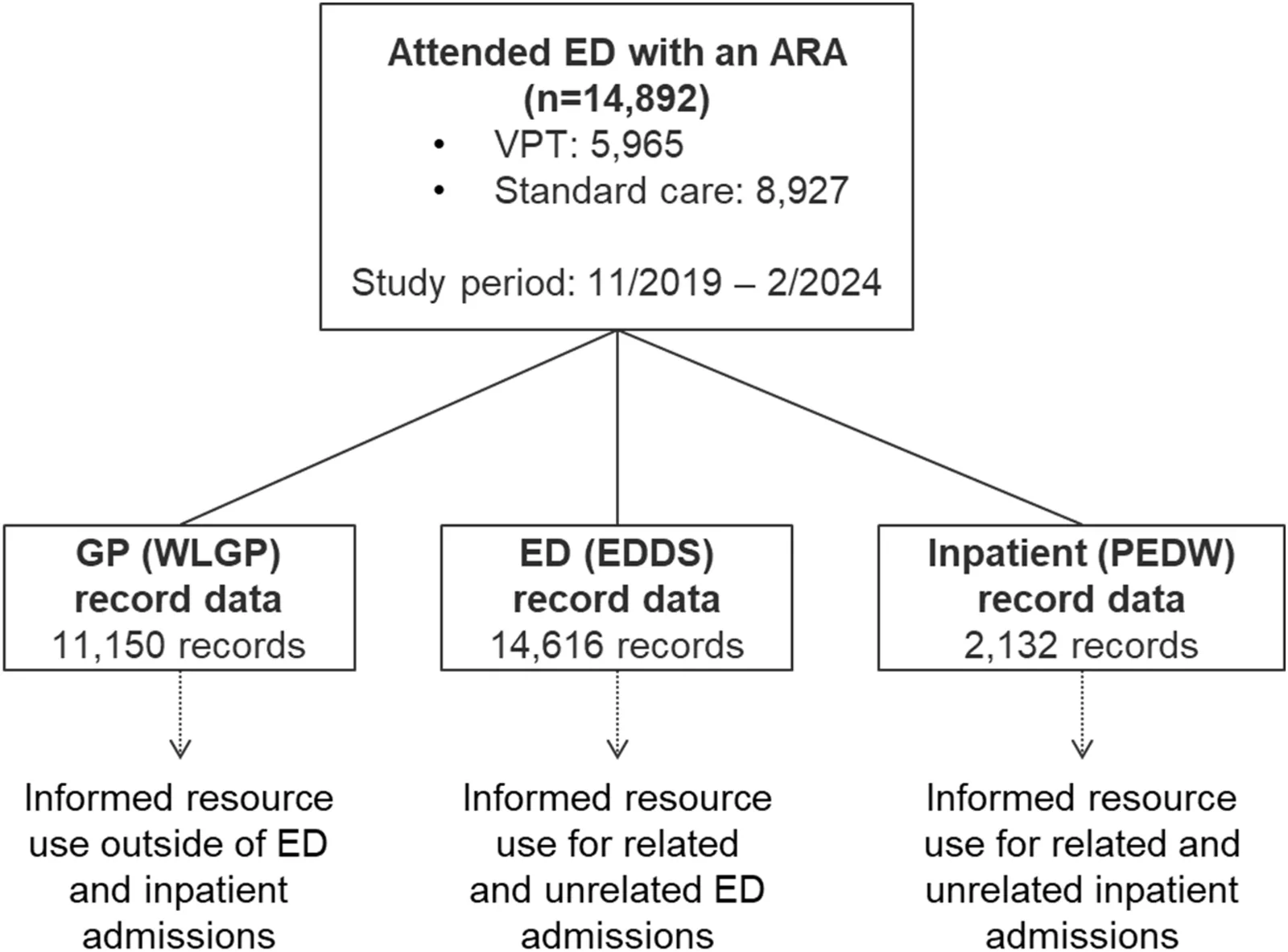
Data linkage. Abbreviations: ARA, assault-related attendance; ED, emergency department; EDDS, Emergency Department Data Set; GP, general practitioner; PEDW, Patient Episode Data for Wales; WLGP, Welsh Longitudinal General Practitioner Dataset.

Individuals who were exposed to the VPT or standard care were identified using their index ED attendance, where those in the standard care group would have attended an ED without a VPT. The research team subsequently confirmed all individuals assigned to the standard care group were not exposed to an intervention ED during their index attendance.^9^ Participants were matched using coarsened exact matching,^16^ with a minimum 1:1 intervention-control allocation ratio, on several socio-demographic characteristics including patient age, sex, ethnicity, residential deprivation, urban/rural classification, time to inclusion, hospital admission status, and high intensity user status.^9^ Details on how each variable was defined and categorised are provided in Supplementary Appendix A.3. Data linkage, cleaning, and analysis took place within the secure SAIL environment using SQL, SPSS syntax, and Stata 18. Following data linkage and cleaning, the sample consisted of 5965 individuals offered a VPT assessment and 8927 individuals that were not (Fig. 1). We then linked to individuals’ GP, ED, and inpatient records to estimate mean probabilities, resource use and costs for the VPT and standard care groups.

### Cost-effectiveness

**Fig. 2:**
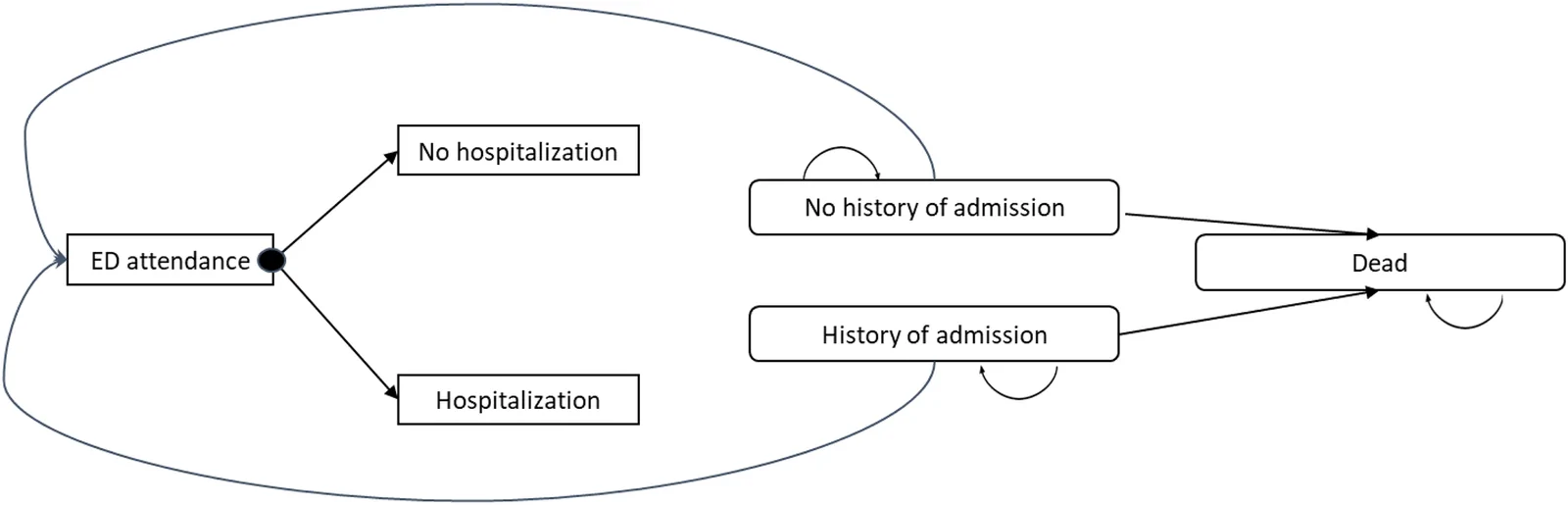
Simplified schematic of decision analytic model. Abbreviation: ED, emergency department.

Following a review of published decision analytic models evaluating the cost-effectiveness of violence injury services and road traffic accidents resulting in injury, we opted to develop a *de novo* hybrid decision analytic model in R to capture both the short- and long-term consequences of assault-related attendances (Fig. 2). This model structure differs from what has previously been used in the literature^13^ by including a long-term (10 year) time horizon and by factoring in patients' history of admission.

The model begins with individuals who attended emergency services for an assault-related event. Patients could be admitted into hospital or discharged from ED without hospital admission. For those who are admitted, they are classified as having a history of hospital admission. Following treatment, patients entered a natural history Markov model with history of admission or no history of admission. They remained in their current health state or re-entered the decision tree via a subsequent unplanned ED attendance, whereby they may be admitted and subsequently classed as having a history of admission. Patients also remained at risk of all-cause death throughout the model. Given no short-term rates of mortality in our sample, we did not capture acute mortality associated with the assault or differences based on history of admission in the long-term. Quarterly cycles were applied with a time horizon of 10 years. We implemented a 10-year time horizon to capture a reasonable period over which differences in patient outcomes, resource utilisation, and costs for those exposed to the VPT or standard care may persist. After this time, we assume there are no further differences between groups. Three Markov tunnels were developed to reflect the differing probabilities, costs, and payoffs associated with unplanned ED events as time elapsed (i.e. the model tracked potential history for up to three or more unplanned ED attendances). Costs and benefits beyond the first year were discounted at 3.5% per annum, as recommended by UK guidelines.^17^ The model was run in duplicate for those who were offered assessment with VPT and those who were not, with probabilities and costs differing by group.

### Probabilities

Model probabilities included the proportion of individuals that were hospitalised (i.e. admitted overnight) following their ED attendance and the probability of subsequent unplanned ED attendance. Probabilities were adjusted by baseline patient characteristics using logit regression models (see Supplementary Appendix A.3 for model covariates) and were differentiated by history of hospitalisation and intervention, enabling us to capture heterogeneity in resource use and costs. Table 1 provides additional information on the values used for each probability parameter.

**Table 1:** Base case probability values.

Variable	PSA distribution	Probability
Probability of hospitalisation^a^—standard care	Beta	0.016–0.825
Probability of hospitalisation^a^—VPT	Beta	0.009–0.622
Probability of subsequent unplanned ED visit for those with no history of admission—standard care	Dirichlet	0.018
Probability of subsequent unplanned ED visit for those with no history of admission—VPT	Dirichlet	0.017
Probability of subsequent unplanned ED visit for those with history of admission—standard care	Dirichlet	0.017
Probability of subsequent unplanned ED visit for those with history of admission—VPT	Dirichlet	0.015

**Abbreviations**: ED, emergency department; PSA, probabilistic sensitivity analysis; VPT, Violence Prevention Team.

a Reflects heterogeneity in resource use across subsequent ED visits.

### Resource use and costs

Healthcare resource use was estimated by totalling the number of ED attendances as well as primary and secondary care visits per quarter, which was sourced using routine data from SAIL. The total number of primary and secondary care visits were measured on a quarterly basis over the study follow up period (12–48 months) and converted into costs by applying UK national sources.^18^, ^19^, ^20^ Costs are reported in 2023/2024 British pounds sterling. ED attendances were costed at £273 per visit while GP visits were costed at £77 per visit (£49 consultation cost + £28 prescription cost). Costs associated with hospital admissions were retrieved from inpatient records, where Hospital Resource Group (HRG) reference codes, and their associated costs, were assigned to each hospital spell based on clinically comparable treatments that used similar NHS resources.^20^ Table 2 provides the costs per ED attendance and hospital admissions and mean quarterly and standard error (SE) cost estimates for the VPT and standard care arms.

**Table 2:** Base case cost values (mean total quarterly estimates).

Variable	PSA distribution	Mean	SE
Cost per ED attendance not resulting in hospital admission	–	£273	–
Cost per ED attendance resulting in hospital admission—VPT	Gamma	£1376	£814
Cost per ED attendance resulting in hospital admission—standard care	Gamma	£1001	£674
Cost of GP visits for no history of admission—VPT^a^	Gamma	£5–£20	£42–£54
Cost of GP visits for no history of admission—standard care^a^	Gamma	£18–£58	£35–£44
Cost of GP visits for history of admission—VPT	Gamma	£68	£288
Cost of GP visits for history of admission—standard care	Gamma	£68	£288

**Abbreviations**: ED, emergency department; GP, general practitioner; PSA, probabilistic sensitivity analysis; SE, standard error; VPT, Violence Prevention Team.

a Reflects heterogeneity in resource use across subsequent GP visits.

Cost data are known to be left-skewed with a substantial number of zeroes, and there is presently no central method for analysis.^21^ We estimated the mean quarterly healthcare costs for the VPT and standard care groups using generalised estimating equations (GEE), which are a suitable class of models for use with correlated longitudinal panel data. We tested several potential models and applied the Quasi-Information Criterion (QIC) to determine the most suitable family and link function for each cost variable. Model covariates included age, gender, follow up time, Welsh Index of Multiple Deprivation (WIMD) quintile, urban/rural status, ethnicity, and high intensity user (details are provided in Supplementary Appendix A.3).

We estimated the VPT intervention costs by summing the total salary cost for a mid-Band 6 NHS nurse over the study period (2019–2024), divided by the total number of patients who were provided a VPT assessment. Total VPT cost was estimated as £45 per person, on average.

To capture the societal perspective, we also considered the costs to third sector organisations. Information on referral to these third sector organisations was available for individuals who interacted with VPTs but not for those in the standard care group. Third sector service costs were estimated using a top-down costing approach, where we combined the total operating costs per third sector organisation (accessed through publicly available information) and divided that by the number of service users to estimate a cost per user. This was combined with the information on referrals. Third sector service costs were estimated at £170 per person, on average. We assumed third sector service use in the standard care group was zero.

### Health outcomes

Health outcomes were measured in QALYs and reflected a combination of an individual's HRQoL over time and of health decrements associated with ED attendance (reflecting health shocks caused by injury and the need for admission or not). HRQoL data is commonly measured using generic preference-based self-reported outcome measures, such as the EuroQol 5-Dimension, 5-Level (EQ-5D-5L) measure, which describes quality of life across five domains, with five severity levels for each.^22^ Each state is then converted to a cardinal utility scale, where higher values (to a maximum of 1) represent preferable health states. Values of 1 represent full health, 0 represents death, and those less than 0 represent health states that are considered worse than death.^23^

We estimated QALY decrements for each hospitalisation following an ED visit and accumulated additional HRQoL decrements according to history of no admission and history of admission for each model cycle. Values were sourced from the literature (Table 3).^24^^,^^25^ The HRQoL decrements were applied against the average age- and sex-adjusted HRQoL for individuals in England to capture the health states of the Markov model.^26^

**Table 3:** Quality of life decrements.

Variable	PSA distribution	Value
QALY decrement, no hospitalisation	Gamma	0
QALY decrement, hospitalisation^21^	Gamma	0.07
HRQoL decrement, no history of admission^22^	Gamma	0.16
HRQoL decrement, history of admission^22^	Gamma	0.24

**Abbreviations**: PSA, probabilistic sensitivity analysis; QALY, quality-adjusted life year; HRQoL, health-related quality of life.

### Cost-effectiveness outcomes

The model reports the incremental cost-effectiveness ratio (ICER) as the cost per QALY for those receiving VPT assessment versus standard care, as well as the net health and net monetary benefits for each alternative and probabilities of the VPT being cost-decreasing, QALY-increasing, and cost-effective. ICERs were interpreted against the Department for Health and Social Care's cost-effectiveness threshold of £15,000 per QALY^27^ and the National Institute for Health and Care Excellence (NICE) cost-effectiveness threshold of £20,000–£30,000 per QALY.^28^

### Sensitivity analysis

To address overall uncertainty in the model, we conducted a probabilistic sensitivity analysis (PSA) with 1000 Monte Carlo simulations, where parameters were varied simultaneously based on mean and standard error values using beta, gamma and Dirichlet distributions as appropriate (see Table 1, Table 2, Table 3).^29^

### Scenario analysis

We conducted two scenario analyses. Scenario A captured all ED and inpatient costs but equalised background GP costs for individuals in the VPT and standard care arms. This scenario assumed that the VPT did not impact healthcare costs other than by reducing the number of subsequent ED attendances and the probability of having had an inpatient admission. In Scenario B, we excluded all costs associated with follow-up visits in primary care occuring in between ED attendances and/or hospital admissions. This scenario assumed that only ED and inpatient costs had a material impact on the model.

### Ethics

The SAIL databank is an ISO 27001 certified and UK Statistics Authority accredited secure data environment. Approval to access and analyse data housed in SAIL was granted following independent review by the SAIL Independent Information Governance Review Panel (approval number 1421). As the study uses anonymised data, patient consent was not required.

### Role of the funding source

The funder had no role in study design, data collection and analysis, decision to publish, or preparation of the manuscript.

## Results

Table 4 provides an overview of the sample included for this study. Individuals were on average 30 years of age, the majority (86%) were of White ethnicity, and two-thirds were male. Approximately 40% lived in the most deprived quintile and nearly all (92%) lived in urban communities.

**Table 4:** Sample characteristics.

Variable	Sample (n = 14,892)
Age	N (%)
11–17 years	2687 (18.4)
18–30 years	5798 (38.9)
31–50 years	5098 (34.2)
50+ years	1309 (8.8)
Sex	
Female	5037 (33.8)
Male	9855 (66.2)
WIMD	
1 (Most Deprived Quintile)	6108 (41.0)
2	3646 (24.5)
3	2165 (14.5)
4	1502 (10.1)
5 (Least Deprived Quintile)	1471 (9.9)
Urban/Rural	
Rural	1149 (7.7)
Urban	13743 (92.3)
Ethnicity	
White	12804 (86.5)
Mixed or multiple	147 (1.0)
Asian	385 (2.6)
Black	78 (0.5)
Another ethnic group	1393 (9.4)

**Abbreviation:** WIMD, Wales Index of Multiple Deprivation.

The overall engagement in VPT referrals was 35%. Probabilistic cost-effectiveness results are presented below (deterministic cost-effectiveness results are presented in Supplementary Appendix A.4). Table 5 presents the mean values of the simulated results for both the health and societal perspectives as well as the scenario analyses. In our base case analysis, VPTs in ED were less costly and more effective (dominant) compared to standard care, for both the health and societal perspectives. VPTs in ED also remained cost-effective when primary care costs were equalised (Scenario A) or removed (Scenario B) using a health perspective (£11,820 and £12,950 per QALY gained, respectively). However, from a societal perspective, in Scenarios A and B VPTs were not considered cost-effective.

**Table 5:** Probabilistic cost-effectiveness results.

Perspective	VPT costs	Standard care costs	VPT QALYs	Standard care QALYs	Inc Cost	Inc QALYs	ICER
Base case							
Health	£940	£1643	4.55317	4.54916	−£703	0.00401	Dominant
Societal	£1070	£1642	4.55349	4.54937	−£572	0.00412	Dominant
Scenario A: Primary care costs equalised							
Health	£1677	£1634	4.55339	4.54979	£43	0.00360	£11,820
Societal	£1888	£1687	4.55349	4.55044	£201	0.00305	£65,696
Scenario B: Primary care costs set to £0							
Health	£466	£423	4.55285	4.54955	£43	0.00330	£12,950
Societal	£635	£424	4.55338	4.54910	£211	0.00428	£49,315
Probability VPT cost-effective at £15,000 per QALY threshold^a^							84.1%
Probability VPT cost-effective at £20,000 per QALY threshold^a^							83.8%
Probability VPT cost-effective at £30,000 per QALY threshold^a^							83.3%
Probability VPT is cost-saving (cost-decreasing)^a^							83.7%
Probability VPT is more effective (QALY-increasing)^a^							65.1%

**Abbreviations:** Inc, incremental; ICER, incremental cost-effectiveness ratio; QALY, quality-adjusted life year; VPT, Violence Prevention Team.

a Health perspective.

**Fig. 3:**
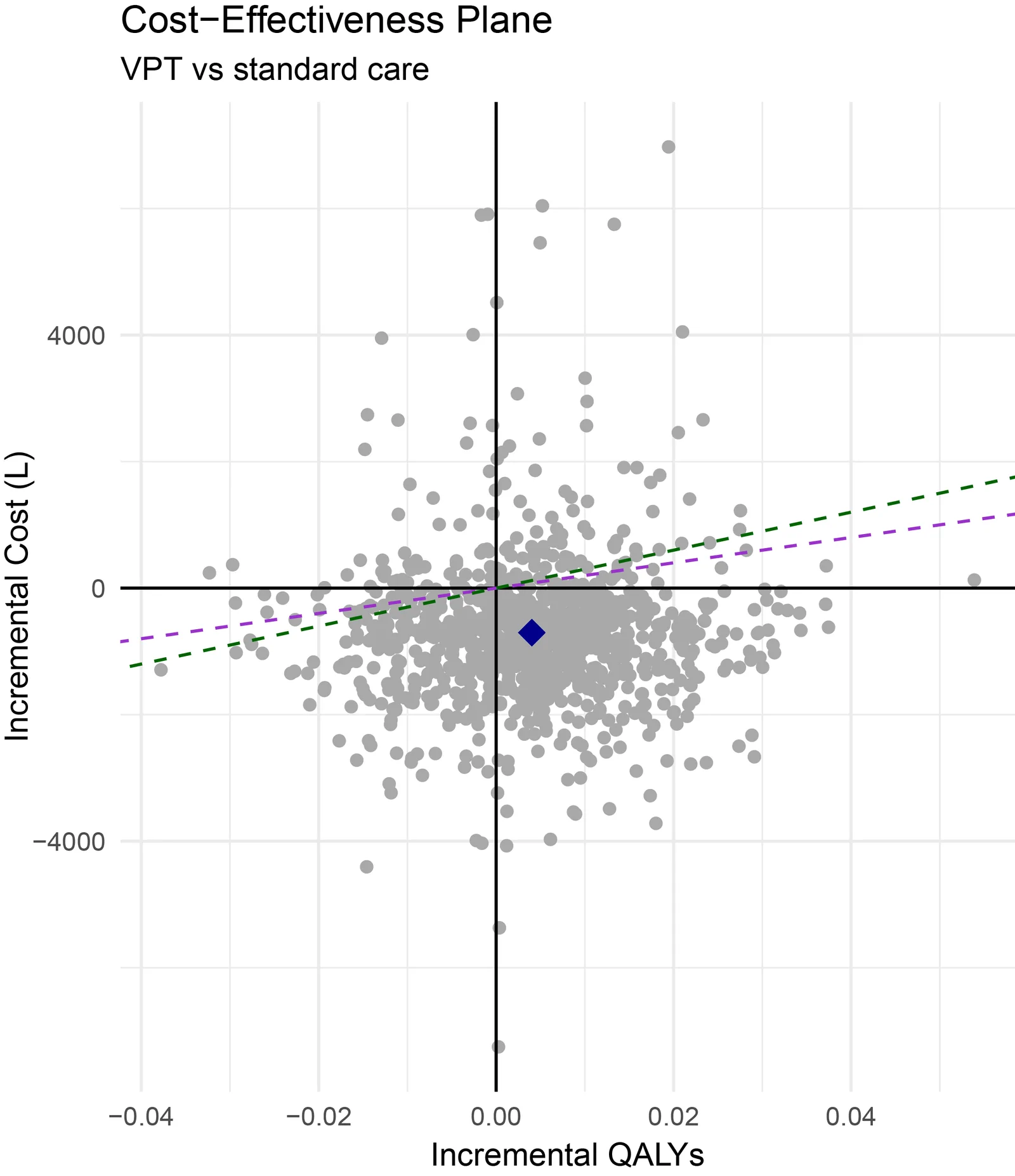
Cost-effectiveness plane (health perspective). Dashed lines represent the NICE £20,000–£30,000 per QALY threshold; grey dots represent results for each iteration of the Monte Carlo simulation; large blue dot represents the mean cost and mean QALY across all simulated results.

Fig. 3 presents the cost-effectiveness plane from a health perspective, where we plot the results for each iteration of the Monte Carlo simulation. More than half the iterations (54%) fall within the ‘cheaper but more effective’ quadrant, suggesting that VPTs in ED are cost-saving and more effective (dominant) relative to standard care. This is visually represented by the large dot in Fig. 3. At a NICE cost-effectiveness threshold of £20,000–£30,000 per QALY (represented by the dashed lines), there is an 83% probability that VPTs in ED are considered cost-effective relative to standard care (Table 5). At a threshold of £15,000 per QALY, the probability that VPTs in ED are considered cost-effective relative to standard care is 84%. Finally, across all iterations, the probability that VPTs in ED are cost-saving is also 84%, while the probability that VPTs are more effective (QALY-increasing) relative to standard care is 65% (Table 5).

Table 6 provides the net monetary and net health benefits associated with VPT and standard care from a health perspective, using varying cost-effectiveness threshold levels. At a threshold of £15,000 per QALY, the incremental net monetary benefit is £764 per person in favour of VPTs, and £783 and £823 in favour of VPTs for thresholds of £20,000 and £30,000 per QALY, respectively. Incremental net health benefits range between 0.03 and 0.05 QALYs per person in favour of VPTs.

**Table 6:** Net monetary and net health benefits.

Threshold	NMB—VPT (£)	NMB—standard care (£)	Incremental NMB (£)	NHB—VPT (QALYs)	NHB—standard care (QALYs)	Incremental NHB (QALYs)
£15,000 per QALY	67,358	66,594	764	4.49	4.44	0.05
£20,000 per QALY	90,123	89,340	783	4.51	4.47	0.04
£30,000 per QALY	135,655	134,832	823	4.52	4.49	0.03

**Abbreviations:** NMB, net monetary benefit; NHB, net health benefit; QALY, quality-adjusted life year; VPT, Violence Prevention Team.

## Discussion

In this study, we aimed to evaluate the cost-effectiveness of implementing VPTs in ED relative to standard care in Wales, from both a health and societal perspective. HVIPs are strategically placed to intervene following an injury, capitalising on a ‘reachable moment’ for those recovering from injury who are exposed to violence.^30^ A previous cost-of-illness study conducted in Wales estimated that approximately 84% of injury-specific short-term health-related costs are associated with interpersonal, rather than self-directed, violence.^3^

Using a *de novo* hybrid decision tree-Markov model, we estimated the ICER for VPTs relative to standard care over a 10-year time horizon. Our base case analysis suggests that implementing VPTs is less costly and more effective relative to standard care. These findings were consistent when including only the smaller sample of individuals who engaged in VPT referrals (not shown). The cost savings we observe is primarily driven by the effectiveness of the VPTs in reducing subsequent unplanned ED attendances and related hospitalisations (hazard ratio: 0.58, 95% confidence interval: 0.46–0.73), as determined in our broader effectiveness study.^10^ The relative decrease in unplanned emergency visits was combined with lower primary and secondary healthcare costs, contributing to an overall cost savings for the NHS, as demonstrated through the current analysis. If the UK were to implement VPTs in ED at scale, assuming current VPT referral engagement rates, the programme would be introduced to approximately 145,000 people^31^ and result in a relative reduction of just over 7500 unplanned ED attendances and a cost savings to the NHS of £102 million. Greater cost savings could be generated if engagement rates for VPT referrals were to increase above 35%.

Although these study findings align with other economic studies, which also found that HVIPs were cost-saving or cost-effective,^32^, ^33^, ^34^ previous studies have largely taken a health perspective.^13^ While our base case results were favourable for VPTs, once primary care costs were equalised (Scenario A) or removed (Scenario B), VPTs in ED were not considered cost-effective using a societal perspective. However, our societal results are subject to two key limitations. First, we were unable to assess the broader benefits across multiple sectors (e.g. education, criminal justice); we only assessed the costs. In one study, incarcerated Indigenous Australians who engaged in a violence prevention program were significantly less likely to commit new violent offences after release from incarceration compared to those not engaged.^35^ Future research should consider the inclusion of additional effectiveness data across multiple sectors. Second, we assumed that third sector service use for those who received standard care was nil, due to data unavailability. This potentially resulted in an overestimation of the ICER, although the true direction of impact remains unknown. Further, while referrals to other sectors (e.g. education and criminal justice) were also included in the VPT referrals list, we did not have sufficient data for these to be able to estimate additional societal costs, which was also a limitation in this study. While our analysis has demonstrated the value of funding VPTs by the NHS, moving forward, there is opportunity to consolidate a multi-agency approach to funding VPTs, as implementation costs are small (£45 per person, on average) while the potential multi-sectoral downstream cost savings are large. Further, to help inform decision makers, a more detailed budget impact assessment of setting up VPTs would be beneficial.

Strengths of this study include the use of population health records to support the identification of model parameters and the use of a model structure that included a longer-term time horizon and factored in history of admission. These elements differ from what has previously been used in the literature^13^ and enabled the inclusion of primary care visits in the analysis. Further, our findings remained robust under conditions of stochasticity, where the probability that VPT was cost-effective at varying cost-effectiveness thresholds was 83–84%, and the probability VPTs were cost-saving was 84% (Table 5). These strong findings add confidence that HVIPs are a good investment in the UK, as they were largely driven by the effectiveness of the VPTs in reducing subsequent unplanned attendances in ED,^10^ contributing to a reduction in primary and secondary healthcare costs. Our analysis also represents patients’ exposure to the VPT service irrespective of their uptake with referrals. These findings highlight opportunities to increase the uptake of VPT referrals beyond 35%, which will result in improved outcomes for patients and greater cost savings for the NHS. Our findings also reflect all injury types and severities. However, due to the limited nature of the routine data used in this study, we were unable to delve further into the details surrounding types of violence. Future research stratifying cost-effectiveness by type of violence will be considered.

In conclusion, VPTs in ED have the potential to reduce primary and secondary healthcare costs and improve health outcomes for patients affected by interpersonal violence, as staff are strategically placed to intervene following injuries. Accordingly, HVIPs should be prioritised by the NHS for implementation.

## Contributors

SCM, AmB, DOR, and SW pursued funding for this study. All authors were involved in the conception or design of the work. SP, SCM, DT, MH, and AmB were involved in the acquisition of data for analysis; SP and SW were involved in the analysis and interpretation of data. SP, SCM, AmB, DT and SW had full access to and verified the data and analytics. SP and SW drafted the initial manuscript. All authors were involved in reviewing the manuscript critically for important intellectual content, provided final approval of the version to be published, and agree to be accountable for all aspects of the work.

## Data sharing statement

As the data used in this study include private medical information it cannot be shared. However, researchers can apply for access to both data and the analytic code used from SAIL (www.saildatabank.com). The economic model code used for this study is available by request from the corresponding author.

## Declaration of interests

SCM reported receiving grants from the Medical Research Council (MRC), National Institute for Health and Care Research (NIHR), Youth Endowment Fund, Office of the South Wales Police and Crime Commissioners Office, outside the submitted work. SCM is Deputy Chair of the NIHR/MRC Better Methods Better Research Funding Panel and received honoraria as Visiting Professor from Università Cattolica Del Sacro Cuore, Milan, Italy.

MH reported receiving grants from the NIHR. AmB reported receiving grants from the EU and UKRI. The institutions affiliated with SCM, AmB, and SW all received funds for the current study. No other competing interests were declared.
