# Supplementary material for: Nurse-led hospital violence intervention programme versus standard care in Wales: a cost-effectiveness analysis
Source: eClinicalMedicine. 2026 Jul 24;98:104108. doi: 10.1016/j.eclinm.2026.104108 (PMC13507667; doi:10.1016/j.eclinm.2026.104108)
Supplement: Supplementary file 1 [file mmc1.docx]

Supplementary Appendix

Nurse-led Hospital Violence Intervention Programme relative to standard care in Wales: a Cost-Effectiveness Analysis

Table of Contents

[A1 Template for Intervention Description and Replication	2](#_Toc233296737)

[A2 VPT Referrals	8](#_Toc233296738)

[A3 Variables included in Coarsened Exact Matching and in regression models	10](#_Toc233296739)

[A4 Deterministic cost-effectiveness analysis results	11](#_Toc233296740)

[References	12](#_Toc233296741)

# A1 Template for Intervention Description and Replication

*Reproduced with permission from the original authors.^1^*

The Template for Intervention Description and Replication (TIDieR) is used to describe the HVIPs evaluated, which was adapted from a formative evaluation of the Cardiff Violence Prevention Team.^2,3^

**Brief Name**

Violence Prevention Team (VPT)

**Preparation of TIDieR**

Preparation of TIDieR was undertaken by

- Cardiff University (Violence Research Group and DECIPHER)
- Youth Endowment Fund (YEF), who also part-funded the intervention and funded the process and implementation evaluation.
- An expert advisory group provided oversight, including experts from Public Health Wales, Welsh Trauma Network, South Wales Police, Office of the Police and Crime Commissioner, and third sector organisations including Welsh Women’s Aid, The Wallich and Black Association of Women Step Out (BAWSO).

**Service Delivery**

- VPT staff (nurse, nurse advocate, community youth workers), who implemented and continually developed the service.
- Clinical teams in the two Emergency Departments (EDs) in which the interventions are situated provide clinical governance.

**Commissioners**

- Wales VPU (partner agencies including South Wales Police Office for the Police and Crime Commissioner and Public Health Wales) provided the bulk of the funding for the interventions and have on-going dialogues regarding the service development, implementation, and delivery at both implementation sites.
- The UK Home Office provided initial funding to the VPU and YEF.

**Intervention Need**

EDs receive for treatment patients who have been exposed to violence. There are numerous reasons why some people are exposed to violence, including their alcohol use, illicit drug use, childhood adversity, relationship violence, coercion or exploitation, amongst other reasons. These risk factors will also be associated with increased utilisation of emergency care generally. EDs therefore have a unique opportunity to offer these patients additional support, beyond treatment for acute health needs, either directly or through referral to other healthcare services, or in discharge planning signposting third-sector organisations, for example. If this support is successful, then the expectation is that patients will exhibit a reduced use of emergency care services in general, not just for violence-related injury.

The primary objectives of the hospital-based service provision are to

a) identify patients attending EDs whose attendance is predicated on their exposure to violence,
b) work with patients to understand any circumstances or risk factors that increase their exposure to violence, and
c) to either support their referral into secondary or third-sector care or to provide ongoing case-management alongside third-sector support.

All patient-facing clinical staff in emergency care, and elsewhere, have a duty of care and will have received training necessary to undertake patient safeguarding. However, the additional resources involved with the VPT afford

a) greater time working with patients to determine need,
b) deepen links with primary, secondary, and tertiary care, and third sector organisations, for improved referral processes,
c) work across clinical teams to support colleagues with their safeguarding needs and referral into the intervention, and
d) through direct contact with patients, support opportunities for disclosure and therefore increase ascertainment of assault-related attendances.

While the VPT can and will refer to any community service provider based on patient need, specific funding has been dedicated to two different organisations where the VPTs are located. The community-based provision provides intensive support to high-risk children and young people (aged 11-24 years) involved in serious organised crime, drug-related activity, and showing signs of exploitation. The objective is to build resilience to enable these young people to be diverted away from further involvement in serious violence and organised crime.

**Physical or Informational Materials Used in the Intervention.**

The VPTs have made resources available to patients and their families, including leaflets providing details of the service, and information packs to inform them of key issues (e.g., county lines and exploitation).

The VPT has available physical and informational materials usually available to ED staff. This includes access to patient records, both specific to the ED and community healthcare generally. This information facilitates risk assessment and safeguarding practices (e.g., identify patterns of attendance at health care settings for violent-related injury), as well as allowing the VPT ensure their engagement with patients is appropriate at that time (with consideration to the patient’s clinical needs).

In addition, the VPT have strong links with South Wales Police, and receive information and intelligence relating to violence related incidents and community problems. The provision of this information and intelligence to the intervention teams can help inform their interactions with patients and ensure the safety of the hospital and patients (e.g., in cases of youth violence where further attempts to harm a patient may occur).

The VPT provide training to a wide range of clinical and non-clinical staff within the hospital, including:

- Clinical hospital staff, on violence (e.g., youth violence) and vulnerability, identifying violence-related injury and engaging with patients, implementing safeguarding procedures for violence-related injuries (e.g., completing multi-agency referral forms, MARFs).
- Reception staff, on data entry and patient coding, to improve the quality of routine clinical data.
- The nature of medical education entails short-term postgraduate and specialty training, and therefore a steady flow of clinical staff new to the ED environment. The VPT provides education and training, and impromptu advice and support through one-on-one interactions, to hospital staff. This includes providing consultation on patients and facilitating patient interactions for more challenging cases.

**The procedures, activities, and/or processes used in the intervention, including any enabling or support activities.**

The VPT engages with patients at different stages of their journey, depending on the type and severity of their injuries, the time and day they attend the ED, and the longevity of their hospital stay. They are embedded in the ED clinical team, and have the same resources (e.g., access to electronic patient management systems) as other ED staff.

Referrals: The VPT can receive patient referrals through multiple channels, including email, phone, and face-to-face contact. During their shift, VPT staff can be notified of eligible patients through a set of questions asked during patient registration, triage or by monitoring the ED patient management system. When the VPT is not on shift, paper-based referral forms are available, and staff can still use written formats to refer patients and the VPT will retrospectively review the ED patient management system to identify any additional patients that may have been missed by clinical staff.

Processes: The VPT provides individualised support to patients based on their needs and collaborates with primary, secondary and tertiary care, third sector organisations and other statutory organisations (e.g., local government safeguarding teams, the police, and school nurses). They establish a relationship with the patient and, where appropriate, their family. They provide emotional and practical support. They also manage risk factors and risks to patients by gathering information and through collaborative multiagency work. This can be through existing resources, such as the Domestic Abuse, Stalking, Harassment and Honour Based Violence Assessment (DASH) tool, and referrals into the Multi Agency Risk Assessment Committee (MARAC).

The VPT will either discharge patients to receive support elsewhere, or, in the case of high risk, high need children exposed to criminal or sexual exploitation, continue their support of the patient alongside third sector organisations specifically funded to support the VPT in this area.

**Access to Information**

The VPT has access to usual healthcare patient management systems, both ED specific and community-based, that can be accessed to inform patient assessments and safeguarding decisions.

Furthermore, the VPT access information on patients to inform risk assessment and management. For example, following incidents of serious violence (e.g., stabbings and shootings), they will gather information on risks and known associations through the police, probation, hospital staff, and through the safeguarding team (who routinely meet to discuss patients).

**Assessment**

The VPT completes a risk- and needs-based assessment in collaboration with patients if they agree to engage and, where appropriate, with their families. This will also include information obtained through patient records (e.g., PARIS and clinical portal) on previous hospital visits.

**Expertise, Background and any Specific Training Given to Each Intervention Provider.**

VPT staff will be trained to Level 2 or Level 3 Safeguarding, available through continuing professional development to all frontline staff in the NHS. VPT staff are typically seconded from the broader ED team and will typically be nurse-led.

Additional third-sector support is funded to provide support to high risk, high need children.

**Modes of Delivery**

The VPT team meets with patients face-to-face in the ED if they are on shift, or on hospital wards if the patient is admitted. If the team is not on shift, or receive referrals from Minor Injury Units, they will follow-up with a phone call and conduct an assessment. All contact is done an individual basis, or in collaboration with the family (for under 16-year-olds, or in cases the patient consents to family involvement).

Under usual safeguarding processes, the clinician who first receives any disclosure is expected to lead on the subsequent referral to the VPT, so that patients are not required to repeatedly describe circumstances they might find distressing. In this latter case, the VPT will support the patient’s referral.

**Location of the Intervention**

The VPT is physically based in the ED. However, they can accept referrals from Minor Injury Units and also provide support to patients on the wards if they are admitted following serious injury. The VPT also works with patients that are transferred from other health care facilities, which is particularly pertinent when the hospital is a major trauma centre, or trauma unit, and therefore accepts out-of-area patients. The VPT engage with other health care settings to facilitate transfers (e.g., provide information on known risks and risk factors), and inform care planning.

**Intervention delivery, number of sessions, schedule, duration, intensity, and dose.**

Patients will either engage with the VPT or will refuse support. If the latter, they will have been in contact with the VPT once. For patients who engage with the VPT, the frequency and duration of engagement with patients will be determined through the patient’s age, clinical need, and any underlying vulnerability.

**Hospital-based VPT**

The VPT typically has one or two interactions with most patients, such as a phone call or text message. However, patients with greater needs may have more frequent contact and remain on the caseload for several weeks. Inpatients are supported until they are discharged. The VPT can only maintain a small caseload of patients who require longer-term support, typically those on waiting lists or who are too vulnerable to disengage with. Patients referred to the caseworker have minimal involvement with hospital-based services.

**Caseworker**

The caseworker offers high-intensity support for high-risk, high-need young people and engages with them two to three times a week. To maintain this level of contact, the caseload is limited to five young people at a time. Some service users may be on the caseload for an extended period if they resist engagement. However, for patients referred to hospital-based services, the caseworker has minimal involvement or contact.

**Intervention Tailoring**

*Level of Harm*

The VPT engages with patients attending ED in consequence of their exposure to violence. All patients are eligible, and initial assessment will determine how patients are managed. In the case of domestic abuse, the patient will be handed over to an Independent Domestic Violence Advocate (IDVA). In the case of sexual abuse, the patient will be handed over to the Independent Sexual Abuse Advocate or the Sexual Assault Referral Clinic. In the case of self-harm, the patients will be handed over Mental Health Services. The VPT will therefore typically engage with patients attending with non-domestic violence-related injuries, of varying acuity. The team offers immediate support to patients with non-life-threatening injuries, while for high acuity patients, they wait until the patient is stable before offering their services. The VPT team conducts assessments for patients admitted into the hospital, which allows them to provide more intensive support.

*Caseworker*

The caseworker engages with patients either in the hospital or in community settings and maintains a caseload of up to five young people at a time for high intensity support.

**Stage of Implementation**

This is an initial TIDieR, generic to two sites at which the VPT model has been implemented. The VPTs at the two sites are at different stages of implementation with one having a significantly longer operational period.

**Assessment of Intervention Fidelity**

The VPT model is subject to a formal evaluation, and a process and implementation evaluation, which this TIDieR informs. The outcome from these evaluations will be a revised logic model, an understanding of the adaptations made to the intervention based on locality, and a formal effectiveness and cost-effectiveness evaluation.

**Actual Intervention Fidelity**

Delivery of the intervention has been impacted by staffing changes in both sites, with both sites operating with reduced staff during periods of their operation. As a result, this has led to delays engaging with and referring some patients for further support.

# A2 VPT Referrals

- Citizens’ Advice: a UK-based organisation aiding people with legal, housing, debt and other services.
- Contextual, Missing, Exploited and Trafficked (CMET): a multi-agency panel for safeguarding.
- Drug and alcohol services led by the NHS.
- Drug and alcohol support given by third sector/ charities, including NEWID drug and alcohol support and BAROD.
- Statutory education: education experiences such as TAITH (International exchange programme).
- Employee wellbeing services, such as the People Health and Wellbeing Service by Cardiff and Vale Health Board for their employees.
- Gender and sexuality support led by third sector organisations, e.g. Umbrella Cymru’s Gender and Sexual Diversity Support Specialists.
- General practitioner.
- Hospital and community care. E.g. outpatient services.
- Housing support given by local authorises such as, houses given by councils, tenancy support units and LA/council-based homelessness support.
- Immigration support from local authorities. E.g., housing given by local councils.
- Independent domestic violence advocates (IDVAs) and independent sexual violence advocates (ISVAs).
- Individuals that can provide support and safeguarding to vulnerable people.
- Inpatient Referral Facility, Facilities with acute care services. May be for drugs and alcohol or mental health.
- Statutory family support: Local Authority/ council-based family services.
- Statutory adult services: Local Authority/ statutory support for adults.
- Letting schools know about support or support from school nurses.
- Local Government support for young people.
- Mental Health support given by NHS organisations, such as Children and Adolescent Mental Health Services (CAMHS) and Adult Mental Health Support Services and Mental Health Liaison Nurses.
- Mental health support given from Local Authorities. Such as, community mental health teams.
- Mental Health support led by third sector organisations such as, MIND, the Amber Project, The Samaritans, Concern Cymru and PLATFFORM.
- Multi-agency referrals and forms, such as, referral into a multi-agency risk assessment conference (MARAC).
- NHS based support for children and young people. Such as, Cardiff and Vale UHB Early Help Services and young people’s/paediatric IDVAs.
- NHS based support for homelessness. Such as, the Cardiff and Vale Homeless Nursing Team.
- Online anonymous crime reporting led by Crime Stoppers. CYP service called Fearless.
- Police reporting with consent, police support, police services and in cases where a weapon is used non-consensual police reporting.
- Primary care support from the NHS. E.g. GPs, pharmacies, dentists and opticians.
- Probation services supporting those who are on probation.
- Local Authority environmental health: public health inspectors who ensure health and safety is upheld in the workplace.
- Sexual Assault Referral Centres (SARC). Supporting those who have experienced sexual assault.
- Single point of access (SPOA): An NHS system where there is a single point of contact where patients can have a clinical assessment by a multidisciplinary team. Patients can be referred on or cases can be held.
- Statutory/ Local Authority safeguarding, such as council-based safeguarding for example, educational safeguarding.
- Support for refugees and asylum seekers, e.g. Oasis.
- Support for victims led by Local Authorities/ statutory bodies. Such as, council-based support for domestic violence.
- Statutory youth support: support from statutory bodies and local authorities such as, Councils Children’s Care Services and Youth services like Cardiff and Vale Early Help Services and Cardiff and Vale Young Wellbeing.
- Violence Prevention Team (VPT) support: support given to patients and cases held.
- Social prescribing: support via community activities based on what the patient enjoys. Such as, boxing and football clubs or gaming.
- Third sector support and treatment for drug-related issues.
- Third sector support for children and young people. E.g. Media Academy Cymru and Action for Children.
- Third sector support for violence-related attendances including domestic violence services. Such as, Victim Focus/Support, New Pathways and St Giles.

# A3 Variables included in Coarsened Exact Matching and in regression models

- Age in years
  - Coarsened exact matching (CEM) across the whole age range used up to 64.9 years, with subsequent categories combined from 65 and over years of age, due to low number in these latter categories.
- Sex
  - Male
  - Female
- Ethnicity, Office for National Statistics five categories
  - Asian: Asian British or Asian Welsh, Bangladeshi
  - Black: Black British, Black Welsh, Caribbean or African, African
  - Mixed: Multiple ethnic groups, White and Asian
  - White: English, Welsh, Scottish, Northern Irish or British
  - Other: Any other ethnic group
- Residence
  - Urban
  - Rural
- Welsh Index of Multiple Deprivation
  - 1 Least Deprived
  - 2
  - 3
  - 4
  - 5 Most Deprived
- Time to inclusion
  - Days, denoting the difference between the date of index assault-related attendance necessary for inclusion in the study cohort and study start date
- Admitted to hospital
  - Admitted to same hospital within local health board (LHB) or admitted to other hospital within LHB or patient dead upon arrival
  - Everything else (including transferred to hospital in different LHB, referred to outpatient department, referred to general practitioner, referred to other healthcare professional, no planned follow-up, planned follow-up at the emergency department, patient self-discharge without clinical consent, died in department)
- High Intensity User
  - Count of emergency department attendances in the preceding month up to index assault-related attendance and inclusion in the study, >=5 were combined into one category due to low numbers.

All continuous variables were coarsened into categorical variables. The number of cuts were per Sturges rule.^4^

# A4 Deterministic cost-effectiveness analysis results

**Table A.3.1: Deterministic results**

| Base case | | | | | | | | | | | | | |
| --- | --- | --- | --- | --- | --- | --- | --- | --- | --- | --- | --- | --- | --- |
| Perspective | VPT  costs | Standard care costs | VPT QALYs | Standard care QALYs | | | Inc Cost | | Inc QALYs | | ICER | |  |
| Health | £907 | £1,620 | 4.55953 | 4.55572 | | | -£713 | | 0.00381 | | Dominant | |  |
| Societal | £1,077 | £1,620 | 4.55953 | 4.55572 | | | -£542 | | 0.00381 | | Dominant | |  |
| Scenario A: primary care costs equalized | | | | | | | | | | | | | |
| Health | £1,705 | £1,666 | 4.55953 | | 4.55572 | £39 | | 0.00381 | | £10,361 | |  |  |
| Societal | £1,875 | £1,666 | 4.55953 | | 4.55572 | £210 | | 0.00381 | | £55,043 | |  |  |
| Scenario B: primary care costs set to £0 | | | | | | | | | | | | | |
| Health | £467 | £426 | 4.55953 | | 4.55572 | £41 | | 0.00381 | | £10,757 | |  |  |
| Societal | £637 | £426 | 4.55953 | | 4.55572 | £211 | | 0.00381 | | £55,439 | |  |  |

**Abbreviations:** Inc, incremental; ICER, incremental cost-effectiveness ratio; QALY, quality-adjusted life year; VPT, violence prevention team

In our base case analysis, VPTs in ED were less costly and more effective (dominant) compared to standard care, for both the health and societal perspective (Table A.2.1). When we equalized background GP costs for individuals in the VPT and standard care arms (Scenario A), VPTs were more costly and more effective relative to standard care. Similarly, when we assumed that only ED and inpatient costs had a material impact on the model and we excluded all primary care costs in the model (Scenario B), VPTs were more costly and more effective relative to standard care. Applying a cost-effectiveness threshold of £20,000-£30,000 per QALY gained, VPTs are considered cost-effective relative to standard care when primary care costs are equalised (Scenario A) or removed (Scenario B) using a health perspective only.

# References

1. Moore SC, Brophy S, Bandyopadhyay A, Hamilton M, Yeoman H, Battaglia A, et al. A quasi-experimental effectiveness evaluation of a nurse-led Hospital Violence Intervention Programme in the United Kingdom using linked routine health and administrative data. eClinicalMedicine. 2026 Apr;94:103848. doi:10.1016/j.eclinm.2026.103848
2. Hoffmann TC, Glasziou PP, Boutron I, et al. Better reporting of interventions: template for intervention description and replication (TIDieR) checklist and guide. *Bmj* 2014;348
3. Newbury A. A service evaluation of the delivery and implementation of a hospital-based Violence Prevention Team within the University Hospital of Wales Cardiff, UK: Public Health Wales; 2022 [Available from: [https://www.violencepreventionwales.co.uk/cms-assets/research/A-Service-Evaluation-of-the-Delivery-and-Implementation-of-a-Hospital-Based-Violence-Prevention-Team-within-the-University-Ho.pdf accessed September 2025](https://www.violencepreventionwales.co.uk/cms-assets/research/A-Service-Evaluation-of-the-Delivery-and-Implementation-of-a-Hospital-Based-Violence-Prevention-Team-within-the-University-Ho.pdf%20accessed%20September%202025).
4. Sturges HA. The choice of a class interval. *Journal of the american statistical association* 1926;21(153):65-66.
